# Conflicting Motor Plans and Sensory Attenuation: Evidence From Event‐Related Potentials for Sounds Generated by Pro‐ and Antisaccades

**DOI:** 10.1111/psyp.70114

**Published:** 2025-07-30

**Authors:** Alexander Seidel, Christian Bellebaum

**Affiliations:** ^1^ Faculty of Mathematics and Natural Sciences Heinrich Heine University Düsseldorf Germany

**Keywords:** antisaccades, auditory ERP, efference copies, saccades

## Abstract

The reduction of neural responses to self‐ versus externally generated stimuli has been ascribed to predictions based on an efference copy of motor commands. However, general predictive mechanisms not specific to movements may also play a role. For antisaccades, that is, eye movements in the opposite direction of a target stimulus, an automated prosaccade has to be suppressed, which may lead to conflicting efference copy signals, as an efference copy is likely created also for the prosaccade. If efference copies for the suppressed and executed saccade are in conflict with each other, prediction mechanisms based on their information are potentially disturbed, which may affect the processing of saccade‐generated stimuli. We compared the N1 and P2 components for pro‐ and antisaccade‐generated sounds with those for visually cued external sounds and found differing temporal dynamics of both components during the course of the experiment, depending on the saccade type. The N1 amplitude for pro‐ but not antisaccade‐generated sounds changed over the course of the experiment, with evidence of an attenuation relative to visually cued sounds at the end. The P2 for prosaccade‐generated sounds decreased already earlier than that for antisaccade‐generated sounds, which only decreased toward the end of the experiment. These findings suggest that both early (N1) and late (P2) processing of saccade‐generated sounds is affected by conflicting efference copies, with the early effect probably reflecting forward model predictions and the later effect indicating agency perception based on these predictions.

## Abstract 2

In this study, we provide evidence for the involvement of motor information in the attenuation of self‐generated compared to visually cued sounds, beyond the involvement of general temporal predictability. We show that only event‐related potential amplitudes for sounds generated by prosaccades, but not antisaccades, decreased significantly over time, likely stemming from conflicting motor signals during antisaccade execution, which demands the suppression of a reflexive prosaccade.

For self‐produced stimuli, studies have consistently shown reduced perceptual intensity and neurophysiological responses compared to externally produced stimuli (Baess et al. 2011; Blakemore et al. 1998; Sato 2008; Schafer and Marcus 1973), referred to as sensory attenuation. In the auditory domain, electroencephalography (EEG) studies typically report reductions of the amplitudes of the event‐related potential (ERP) components N1 and P2 (Baess et al. 2011; Knolle et al. 2013; Sowman et al. 2012), reflecting a neurophysiological sensory attenuation effect. This has been suggested to reflect predictive mechanisms with respect to the sensory consequences of actions, incorporating internal signals and context information to match re‐afferent sensory stimuli with their predictions (for a review, see Horváth 2015). But some studies suggest a functional dissociation between these two components: While the P2 has been shown to be sensitive to contextual factors, like the perceived control (Seidel et al. 2021) and agency (Kühn et al. 2011; Timm et al. 2016) over sound production and is attenuated also for visually cued externally generated sounds (Sowman et al. 2012), the N1 was not affected in these studies. Thus, the attenuation for the two components might rely on predictions based on different types of information.

While the P2 attenuation has been ascribed to general, motor‐independent mechanisms (e.g., Baess et al. 2011; Ghio et al. 2018; Knolle et al. 2013), the prevalent account for explaining the N1 attenuation suggests that cerebellar feedforward models employ efference copies of motor commands to start the generation of predictions concerning their sensory consequences right after the motor planning stage, enabling a matching process with the actual sensory consequences as early as in the N1 time window, around 100 ms after stimulus onset (Blakemore et al. 2001; Horváth 2015; Pickering and Clark 2014; Popa and Ebner 2018; Reznik et al. 2018; Vercillo et al. 2018; Wolpert and Flanagan 2001). This view is supported by the findings obtained in Timm et al. (2014) showing an N1 attenuation only for voluntary actions and not when actions were externally induced via transcranial magnetic stimulation. Examining the supposed cerebellar contribution to the prediction of sensory action consequences, Knolle et al. (2012, 2013) reported a reduced N1 attenuation for cerebellar lesion patients compared to healthy controls, supporting the claim that efference copy‐driven cerebellar forward models underlie the N1 attenuation. At the same time, the P2 was not affected by cerebellar lesions, supporting its independence from cerebellar forward models.

It has been argued, however, that motoric signals are not necessary to generate the predictions underlying the N1 attenuation, because action execution offers sufficient cues for temporal prediction of action‐generated sound onsets, while onsets of external sounds are not predictable (Hughes et al. 2013). In fact, comparing cued self‐ and externally generated sounds, and thus matching temporal predictability, Kaiser and Schütz‐Bosbach (2018) and Harrison et al. (2021) found no (additional) N1 attenuation for self‐produced sounds. Klaffehn et al. (2019) on the other hand reported an N1 attenuation for self‐produced sounds even when controlling for temporal predictability, suggesting that motor‐specific N1 attenuation effects may exist beyond unspecific prediction effects.

Dogge et al. (2019) also argued that forward models are unlikely to underlie N1 attenuation for environment‐related predictions such as sounds following button presses (as opposed to body‐related prediction such as when touching your left hand with your right hand), which are used in most studies, as work in animals has revealed that the tuning of motor‐based forward models is quite slow and studies typically entail only short training. Button presses such as on computer keyboards or phones are, however, likely commonplace in the everyday life of most humans, and forward models are probably well trained for their auditory feedback. In an experimental setting, learning new, specific button press‐sound associations may thus require only minimal training. The study by Mifsud et al. (2016) suggested that experience with specific associations between actions and their sensory effects indeed plays a role. They reported smaller N1 attenuation when sounds were produced by saccadic eye movements compared to button presses, which are typically not associated with auditory consequences in everyday life. At the same time, this study shows that even for unusual action‐sensory effect combinations, the N1 attenuation can be found.

Moreover, single trial‐based linear mixed effects analyses allow us to explore the temporal dynamics of ERP amplitude changes over the course of an experiment by adding the trial number as a predictor (Volpert‐Esmond et al. 2021), thereby modeling effects of practice or experience. Applying this technique thus allows us to model training or experience effects. In a study on sensory attenuation in action observation, we found a change in N1 attenuation over time for an uncommon first‐person observer viewpoint, but not for a common third‐person viewpoint (Seidel et al. 2023). It is thus conceivable that similar temporal dynamics emerge for uncommon action‐sensory effect associations such as sound‐generating saccadic eye movements.

In the present study, we explore the temporal dynamics of N1 and P2 amplitude attenuation for sounds following saccadic eye movements and thus for an untrained action–effect association. In addition, we explore the role of conflicting efference copy signals on N1 and P2 attenuation and their temporal dynamics by comparing the processing of sounds generated by anti‐ and prosaccades. The two conditions are comparable in their motor requirements, but differ in the motor planning and possibly the relayed efference copies. For (pro)saccade‐generated sounds, we expect a significant N1 attenuation (see Mifsud et al. 2016), which becomes stronger over time with increasing experience, as the forward model needs to be tuned for this unusual action‐sensory effect association. For correct antisaccade execution, it is assumed that a motor plan for an automatically planned, but not executed saccade to the appearing target competes with the motor plan of the antisaccade (Coe and Munoz 2017; Munoz and Everling 2004). There is evidence that even in the absence of action execution such as in motor imagery for touch (Kilteni et al. 2018) or inner speech (Jack et al. 2019; Whitford et al. 2017), an efference copy is elicited, leading to N1 attenuation. We therefore speculate that for the motor plan of the automatically planned, but not executed saccade toward the target, an efference copy could still be generated and create a conflict with the efference copy for the executed antisaccade, also with respect to their sensory consequences, which differed between both actions. This should result in a disruption of the proposed cerebellar feedforward model relying on this signal, which is expected to affect sensory attenuation as reflected in the N1 amplitude. Consequently, the attenuation of the N1 amplitude is expected to be stronger for pro‐ than for antisaccades.

As the P2 is independent from cerebellar forward models (Knolle et al. 2012, 2013), we assumed neither an influence of efference copy‐based predictive mechanisms nor any temporal dynamics. Considering that Mifsud et al. (2016) did not find a P2 attenuation for saccade‐generated sounds, we expected neither a general amplitude reduction for saccade‐generated compared to externally generated sounds, nor differences in P2 amplitudes between pro‐ and antisaccade‐generated sounds or amplitude changes over time.

## 1 Method

### 1.1 Participants

Thirty‐eight participants (25 women, mean age 24.8 years, SD = 4.5 years) took part in this study for either course credit or monetary compensation. The sample size was larger than in some previous studies on button press‐elicited sounds (Baess et al. 2011; Ghio et al. 2021, 2018; Klaffehn et al. 2019), but comparable to the study by Mifsud et al. (2016), who first reported an N1 attenuation for saccade‐elicited sounds and tested 36 participants. As we expected similar effect sizes in our study, we aimed for a comparable sample size. However, fifteen participants had to be excluded from data analysis, as too few trials remained for the analysis of the EEG data, mostly because the performed saccades did not fulfill the inclusion criteria (see below for details). The dataset entering statistical analysis thus consisted of 23 participants (13 women, mean age 24.2 years, SD = 3.8 years).

All participants reported normal hearing and normal or corrected‐to‐normal vision, as well as no history of neurological or mental illness, or use of medication affecting the nervous system. All but three participants were right‐handed. Written informed consent was given by all participants before participation. The study was approved by the Ethics Committee of the Faculty of Mathematics and Natural Sciences at Heinrich Heine University Düsseldorf, Germany.

### 1.2 Experimental Paradigm

#### 1.2.1 Stimuli

In all conditions, including the trainings, three black rings with a diameter of 2° visual angle (40 px/° visual angle) and a line thickness of approximately 0.1° visual angle were continuously displayed on the screen on a gray background (R = G = B = 191) to indicate the potential locations in which the target stimuli (see below) could appear during the experiment. This was done to enable easier fixation of target positions after target disappearance and prevent unwanted eye movements during the following time window for ERP analysis. One ring was positioned at the center, the others each 10° visual angle to the left and right. All described target and fixation dots appeared in the left, central, or right ring. Black dot stimuli with a diameter of 1° visual angle were used as visual target stimuli in all conditions of the experiment and in the accuracy check during calibrations. A 1000 Hz sinus tone with a duration of 200 ms (fade in/out of 20 ms) was used as the self‐ and externally generated sound in the different experimental conditions.

#### 1.2.2 Experimental Conditions

In this study, we adapted the classic contingent paradigm (Horváth 2015), in a similar way as Mifsud et al. (2016), by letting participants produce sounds by saccadic eye movements. In contrast to the study by Mifsud et al. (2016), our main interest was in the comparison of sounds elicited by visually guided (pro)saccades toward a visual target and sounds elicited by antisaccades, which are directed away from a target stimulus. The paradigm thus entailed two conditions in which sounds were produced by actions (act‐sound conditions), one with pro‐ and one with antisaccades. As both types of saccades are performed as a reaction to an appearing visual cue stimulus, the conditions with external sounds, which were not associated with any movement, entailed identical cue stimuli to control for effects of predictability based on visual cues (cue‐sound conditions). Importantly, the relative timing of visual cue to saccade, and thus to sound onset, in the act‐sound conditions depended on saccade latency. As antisaccades typically have longer latencies than prosaccades (Munoz and Everling 2004), the paradigm contained two separate cue‐sound conditions, one in which the relative timing of cue and sound was matched to the act‐sound condition with prosaccades and one in which the timing was matched to the act‐sound condition with antisaccades. The cue‐sound condition did thus not serve to provide a perfect control for sound predictability in the act‐sound conditions, but it aimed to control for non‐motor predictions based on the visual cue that might be used in the act‐sound conditions.

**FIGURE 1:**
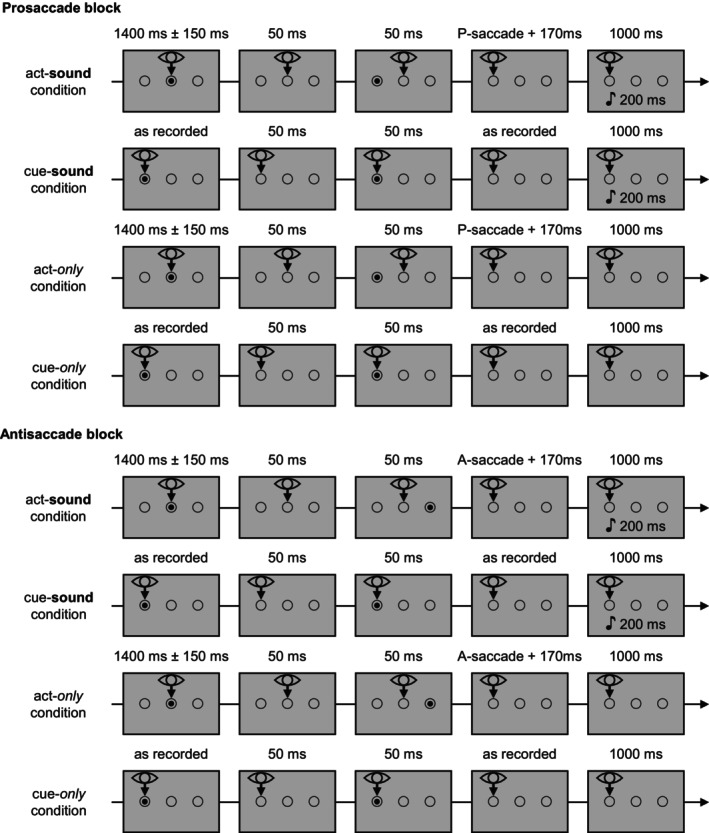
Experimental sequence in the different conditions of one pro‐ and one antisaccade block. Cue‐sound and cue‐only conditions used timings and target stimulus positions as they were recorded in the act‐sound condition at the beginning of each block. In antisaccade blocks, the target position was recorded as the side the antisaccade was aimed at, not the displayed stimulus position. In the act‐sound conditions, sounds were only played if a saccade in the correct direction was detected, and this was replicated in the corresponding cue‐sound condition as well. The order of conditions in each block was fixed.

In addition to these experimental conditions, act‐only conditions were also used in the present study, in which the same movements were performed as in the act‐sound conditions without producing sounds, to correct for motion‐induced ERPs in the act‐sound condition, one with pro‐ and one with antisaccades (see Horváth 2015 for a description of the rationale of the act‐only condition in the contingent paradigm). Accordingly, we also included conditions to control for the visual stimulation in the cue‐sound conditions. In these so‐called cue‐only conditions, a visual cue was shown without presenting sounds (see Figure 1 for an overview of all conditions).

##### 1.2.2.1 Act‐Sound Condition for Prosaccades

Each trial started with a fixation dot in the central ring for 1400 ms (±150 ms random variance, counterbalanced). 50 ms after the fixation dot had disappeared from the screen, a target dot appeared in the left or right ring (counterbalanced per condition) for 50 ms. Participants were instructed to fixate the location of each appearing dot and not move their gaze until the next dot appeared, and were informed that performed saccades to the left or right dot position would cause a sound. During the experiment, eye position was continuously monitored and processed by the program for stimulus presentation. 170 ms after the horizontal eye position reached the position halfway between the fixation point and saccade target position (see below for details), but only if the saccade was aimed in the correct direction, thus only if a prosaccade toward the target dot was performed, a 200 ms sound was played. Reaching the position between the fixation point and target was considered as indicating a saccadic eye movement. The delay ensured that the saccade could be completed before the sound was played. Following this, there was another delay of 800 ms until the start of the next trial. If no sound was played, the timing from the detection of the crossing of the halfway point to the start of the next trial was identical to trials with sound presentation (170 + 200 + 800 ms).

##### 1.2.2.2 Act‐Only Condition for Prosaccades

The act‐only condition was identical to the act‐sound condition, but neither correct nor incorrect saccades caused a sound. Before the condition started, participants were instructed accordingly.

##### 1.2.2.3 Act‐Sound Condition for Antisaccades

The visual stimulation in the antisaccade version of the act‐sound condition was identical to that of the prosaccade version, but participants were instructed to aim their gaze at the ring opposite to the one in which the target dot appeared. Only if correct antisaccades in accordance with the instruction were detected, sounds were presented time‐locked to the antisaccade. In case of a wrong prosaccade, no sound was played. As in the prosaccade condition, sounds were elicited 170 ms after the eye position reached the position halfway between the fixation point and the target position. Note that the target position was on the opposite side relative to the visual target in the act‐sound condition for antisaccades.

##### 1.2.2.4 Act‐Only Condition for Antisaccades

The act‐only condition for antisaccades was identical to the act‐sound condition for antisaccades, but no sounds were presented. Before the condition started, participants were instructed accordingly.

##### 1.2.2.5 Cue‐Sound Condition for Prosaccades

To ensure comparability between act‐ and cue‐sound conditions, the visual stimulation in the cue‐sound conditions was kept as similar as possible to the two act‐sound conditions described above, while not requiring saccadic eye movements. Stimulus timing in each trial of the cue‐sound condition, that is, the duration of fixation dot presentation, visual cue onset, and sound onset time relative to trial start, was determined by the timing of these events in the corresponding trial in the previous act‐sound condition for prosaccades. The cue‐sound condition for prosaccades can thus be considered as a replay of the preceding act‐sound condition for prosaccades. Trials started with the fixation dot. In contrast to the act‐sound conditions, the fixation dot was presented in the left or right ring where also the visual cue would appear later during the trial, depending on the intended saccade direction of the corresponding trial in the previous act‐sound condition. This was done to provide the same visual stimulation as in the act‐sound condition, without inducing automated saccades, and to ensure that eye position during sound presentation was the same in the act‐ and cue‐sound conditions. 50 ms after the fixation dot disappeared, an identical target dot appeared in the same circle as the fixation dot for 50 ms. After a delay depending on the time between target and sound, and thus on saccade latency, in the corresponding trial in the previous act‐sound condition, a 200 ms sound was presented, followed by the next trial 800 ms later. Participants were instructed to fixate dot stimuli as they appeared and focus their gaze on the position until the presentation of the next dot. They were also informed that no saccades were required in this condition. As the cue‐sound condition was a replay of the preceding act‐sound condition, no sounds were played if the participant had made a saccade direction error in the corresponding trial of the act‐sound condition. Instead, the time interval until the next trial started was 1000 ms, to account for the sound duration and keep trial timing consistent, as in the act‐sound condition.

##### 1.2.2.6 Cue‐Sound Condition for Antisaccades

The cue‐sound conditions for antisaccades only differed from the one for prosaccades with respect to the data on which trial timings, target positions, and sound presentation was based. As was pointed out above, we expected both higher error rates and longer latencies for antisaccades, which would be reflected in more sound omissions and longer cue‐sound intervals for the cue‐sound condition for antisaccades compared to prosaccades. The cue‐sound condition for antisaccades was therefore based on the preceding antisaccade act‐sound condition in the same block. Fixation and target dot position in a given trial in the antisaccade version of the cue‐sound condition were determined by the required saccade direction in the corresponding trial of the preceding act‐sound condition for antisaccades. If, for example, participants in trial *x* of the act‐sound condition for antisaccades were required to look to the left (because the target dot appeared on the right), then fixation and target dot in trial *x* of the cue‐sound condition for antisaccades appeared on the left.

##### 1.2.2.7 Cue‐Only Condition for Prosaccades

The visual stimulation in the cue‐only condition for prosaccades was identical to the visual stimulation in the preceding prosaccade cue‐sound condition, including stimulus timings and positions (as recorded in the preceding act‐sound condition). Importantly, no sounds were played in this condition, and participants were informed that no sounds would occur.

##### 1.2.2.8 Cue‐Only Condition for Antisaccades

As with the cue‐sound condition, the cue‐only conditions for pro‐ and antisaccades only differed with respect to the data on which trial timings, stimulus position, and sound presentation was based. The visual stimulation in the cue‐only condition for antisaccades was thus identical to the visual stimulation in the preceding antisaccade cue‐sound condition, including stimulus timings and positions (as recorded in the preceding act‐sound condition for antisaccades). No sounds were played in this condition, and participants were informed that no sounds would occur.

##### 1.2.2.9 Contingency Training

Before the experimental conditions started, participants underwent a two‐part training to establish an act‐sound contingency between saccades and sounds. In the first part, the three circles were presented together with a fixation dot in the center circle for 1500 ms. After an additional delay of 1500 ms, a target dot was presented randomly in the left or right circle for 50 ms, and participants were asked to perform a saccade to the location of the target dot. As soon as a saccade was detected (see information on saccade detection below), a sound was played with a delay of 170 ms. 2000 ms later, the next target dot was shown in the opposite circle, and participants performed another saccade, which caused another sound. This was repeated until 10 sounds had been produced in this manner.

The second part of the training started in the same way as the first part, but after the presentation of the fixation dot ended, participants were instructed to alternate their gaze between the two lateral circles in a rhythm of their choice. Each saccade from the left circle to the right or vice versa prompted a sound with a 170 ms delay, and participants listened to the sounds their saccades produced. No further target dots were shown. The second part of the training ended after 50 sounds had been produced.

### 1.3 Procedure

After signing the consent form and completing the demographic questionnaire, the participants started with the two training tasks to establish a saccade‐sound contingency, followed by short versions of the act‐sound condition (20 trials), once requiring pro‐ and once antisaccades. If participants used visual aids, they removed them before the tasks to improve eye tracking. Participation was only possible, however, when they confirmed that the stimuli during calibrations and tasks were perceptible. The main experiment consisted of four experimental blocks, each containing 40‐trial versions of the four conditions act sounds, cue sounds, act only, and cue only in this fixed order. Each block was either a pro‐ or antisaccade block; that is, it either contained the pro‐ or antisaccade versions of these conditions. The four blocks alternated between pro‐ and antisaccade blocks, and the type of the first block was counterbalanced between participants. Because of this, the first and the second half of the experiment contained one block of each pro‐ or antisaccade condition, and the experimental halves were entered as separate runs into the analysis (see below). To improve performance and minimize the loss of trials, each block started with a short 10‐trial training version of the upcoming act‐sound condition with the respective saccade type. Every block started with a pictured instruction of the upcoming condition. Via button press, the participant could start the block, providing the option for a self‐administered break. The act‐sound and act‐only conditions, in which saccades were assessed, additionally started with a nine‐point calibration of the eye tracker, followed by a short accuracy check.

The experiment was performed using Presentation software (Version 20.3, Neurobehavioral Systems Inc. Berkeley, USA) on a Windows 10 desktop computer and a 22″ monitor with a resolution of 1680 × 1050 px. Sounds were presented via a Sound Blaster Audigy Rx (Creative Technology Ltd., Singapore) using bit accurate playback connected to Sennheiser HD 201 headphones in Presentation's exclusive sound mode at a fixed volume. EEG sound markers and the sound signal were sent with a timing difference consistently measured below 1 ms with a Tektronix TDS 210 oscilloscope (Tektronix Inc. Beaverton, USA).

### 1.4 Online Eye Tracking Data Capture and Analysis

A SensoMotoric Instruments Red 500 eye tracking system (using dark pupil tracking at 500 Hz) was mounted underneath the monitor used for stimulus presentation and connected to a Windows 7 laptop running iView X (Version 2.8.43, SensoMotoric Instruments, Teltow, Germany). A chin rest was used to position participants at a distance of approximately 62 cm from the screen, which resulted in 40 px per degree visual angle.

Saccade detection in all conditions was implemented as a continuous online check of the measured gaze position. A saccade to a left or right target was considered as detected if the *x*‐axis coordinate of the gaze position was further than 5° visual angle (half the distance to a left or right target) from the center of the screen for five consecutive measurements, corresponding to 10 ms. This means that saccades in the wrong direction were detected as well (but counted as an error, see below), except for the contingency training, in which the saccade detection process waited until a saccade in the correct direction was made. In the contingency training, a saccade was considered as detected if five consecutive gaze positions were found in the half of the screen opposite to the last focused circle.

### 1.5 Offline Saccade Detection and Trial Exclusion

From all saccades detected in the raw data with a peak velocity threshold of 40° viewing angle per second (located between 10% and 90% of the saccade length) by the IDF Event Detector (Version 3.0.20, SensoMotoric Instruments, Teltow, Germany), we chose one for each trial that took place during the recorded time of the online saccade detection. For some trials, no matching saccade detected by the IDF Event Detector could be found, and these trials were excluded from the EEG analysis. We then set inclusion criteria concerning saccade features for the remaining trials: As the optimal saccade to a target dot in each trial would have had a length of 10° visual angle, we only accepted trials in which saccade length was between 5° and 15° visual angle, and the saccade starting point was not further than 7.5° visual angle away from the center of the screen. Furthermore, trials were excluded if the sound playback was < 100 ms after the end of the detected saccade, as in this case motor activity would have fallen into the time window of the baseline correction performed during EEG preprocessing.

### 1.6 EEG Data Recording and Processing

Twenty‐eight Ag/AgCl passive ring electrodes were used to record EEG data continuously at 1000 Hz with BrainVision Recorder software (1.21.0402) and a BrainAmp amplifier (Brain Products, GmbH, Germany). An elastic cap (EasyCap, Brain Products) was used for positioning according to the international 10–20 System at F7, F3, Fz, F4, F8, FT7, FC3, FCz, FC4, FT8, T7, C3, Cz, C4, T8, CP3, CPz, CP4, P7, P3, Pz, P4, P8, PO7, PO3, POz, PO4, and PO8. The signal at linked mastoids was used for signal referencing, with a ground electrode at AFz. Horizontal eye movements were recorded at F9 and F10, vertical eye movements at Fp2, and the corresponding position below the right eye. Impedances were kept below 5 kΩ.

Data preprocessing was conducted with Brain Vision Analyzer 2.2.0.7383 (Brain Products). A global direct current de‐trend, a Butterworth zero phase filter (low cutoff: 0.3 Hz, order 4; high cutoff: 30 Hz, order 34) and a notch filter (50 Hz) were applied. By means of an independent component analysis (ICA, steps = 512, infomax restricted biased) components corresponding to blinks in the Electrooculogram channels were excluded before applying an inverted ICA.

Markers for sounds were used to segment data into 800 ms epochs from −200 to 600 ms after sound onset. The conditions in which no sound was played also contained a sound marker, as the sound was muted in these trials. The segmentation could thus be performed in the same way as for the conditions with sounds. After a baseline correction (−100 to 0 ms), the automatic artifact rejection of Brain Vision Analyzer 2.2 was employed to reject noisy segments, with the following parameters: maximal allowed voltage step = 50 μV/ms, maximal allowed difference of values within 100 ms intervals = 100 μV, maximal/minimal allowed amplitude = ±100 μV, lowest activity of 0.5 μV within 100 ms intervals. Act‐sound (and the corresponding cue sound and cue only) segments for trials with erroneous saccades, in which no sounds were played (see above), were not considered for the analysis, as well as trials with erroneous saccades in the act‐only condition. To prevent a large variance in inter‐sound intervals, trials that directly followed trials without sound were removed for the act‐ and cue‐sound condition as well.

Continuing in MATLAB (R2018a, The MathWorks Inc., Natick, MA), we excluded trials in which saccades did not fulfill the inclusion criteria (see above). We subsequently corrected act‐sound and cue‐sound segments for their motoric and visual activity. This is usually done on averaged data, by subtracting the average signal of a motor condition from the relevant average signal in the sound production condition. But to enable further single‐trial analyses of our data, we used an averaged correction signal (from the act‐only and cue‐only condition) and subtracted this from each single segment of the act‐sound/cue‐sound condition, similar to Seidel et al. (2023). However, as we cannot exclude that the ERPs in the control conditions also show a temporal dynamic, we took the potential temporal dynamic in these correction signals into account by performing this procedure not only separately for each block and saccade type for each participant but for three different time bins within each block. For this, we separately averaged act‐only and cue‐only segments for bins consisting of trials 1–13, 14–27, 28–40 in each block and subtracted the averaged signals of the trials in each bin from each single corresponding act‐sound and cue‐sound segment from the same bin within a block, for the respective condition. In the following, the terms act‐ and cue‐sound ERPs will refer to these corrected ERPs. This procedure resulted in the removal of 13 participants, for whom there were less than three trials in at least one bin of the act‐only condition. We furthermore excluded two participants with only one or no trials remaining in one block for one condition in one saccade type.

**FIGURE 2:**
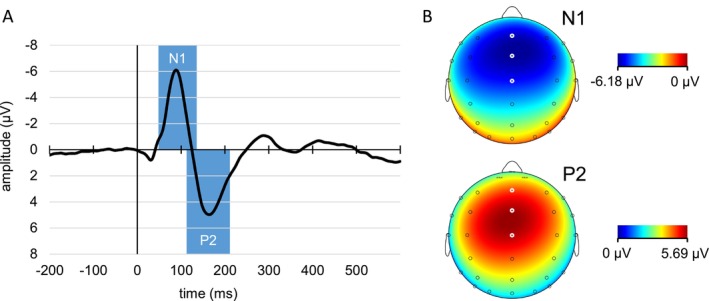
(A) Overall sound‐related grand average ERPs (from all act‐ and cue‐sound conditions) pooled over electrodes Fz, FCz, and Cz. Blue bars show the time windows used to locate participant‐specific peaks in each condition. (B) Topographical maps of scalp potentials at the time of the N1 and P2 peaks from the overall grand average ERPs in A. Electrode positions Fz, FCz, and Cz (from top to bottom) are marked in white.

**FIGURE 3:**
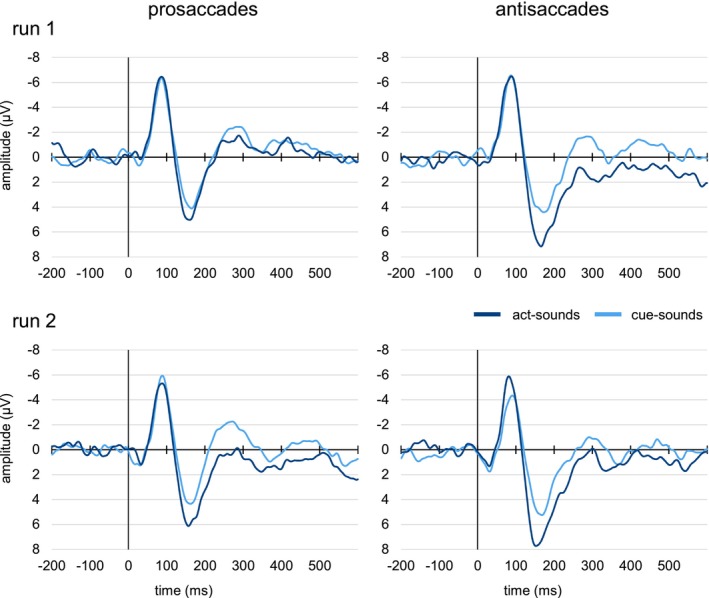
Grand average ERPs for act sounds and cue sounds, grouped by saccade type and run, pooled over electrodes Fz, FCz, and Cz.

To extract single‐trial amplitudes for the components of interest, we first localized the peaks of the N1 and P2 in the overall grand average signal (see Figure 2) pooled over Fz, FCz, and Cz, for which all remaining trials from the act‐ and cue‐sound conditions over all participants were averaged. The mean N1 and P2 peak latencies were determined as 87 and 162 ms, respectively. For each component, we then used a 100 ms time window around these peaks (N1: 37–136 ms, P2: 112–211) to determine participant‐specific peaks in the averaged signal (pooled over Fz, FCz and Cz) for each condition, separately for each saccade type and run (for grand averages see Figure 3, corresponding topographies are shown in Supporting Information S1 and S2). To avoid that very early additional negative peaks were scored as N1 as they occurred in some participants, the N1 window was shortened (48–136 ms) in a second step. An analysis of the peak latency values collected during this step did not show any significant effects or interactions of the factors Sound Type (cue sounds, act sounds), Saccade Type (prosaccades, antisaccades) or Run (first, second) (see Supporting Information S3). In the last step, we collected mean amplitudes in a 40 ms time window around these peaks in the corresponding single‐trial data, resulting in three mean amplitude values per trial (one per electrode) for each component. To remove intrasubject extreme values for each condition within each participant, we excluded single‐trial mean amplitude values deviating more than 2.5 SD from the mean, separately for every participant/electrode/condition/saccade type/run combination.

### 1.7 Statistical Analysis

#### 1.7.1 EEG Data

Components N1 and P2 were analyzed separately, by fitting the amplitude data for each to the same linear mixed effects model. This model included the predictors Sound Type (cue sounds [−0.5], act sounds [0.5]) and Saccade Type (prosaccades [−0.5], antisaccades [0.5]) as the experimental factors. Similar to Seidel et al. (2023), and following suggestions by Volpert‐Esmond et al. (2021), we modeled the course of time over the experiment, employing two further variables. The predictor Run (first [−0.5], second [0.5]) was used to differentiate data from the blocks in the first half of the experiment from those in the second half, while Trial number (1–40) accounted for the order of trials in each run. Instead of numbering the available trials per run and condition after exclusions of error trials and EEG artifacts, this latter predictor contained the original trial numbers before exclusions, to retain the accurate temporal position of each trial. Since missing trials would cause the shifting of the mean when centering this continuous predictor, centering was done using the theoretical mean of 20.5. The model also included all interactions between the four predictors. For random effects, we included a random intercept and random slopes (Sound Type, Saccade Type, Run and all their interactions) for the participants, and additionally a random intercept for the electrodes. The final model formula was:

$$\begin{array}{c} \text{Mean amplitude}\sim \text{Sound type}*\text{Saccade type}*\mathrm{Run}*\text{Trialnumber}\\ +\;\left(1+\text{Sound type}*\text{Saccade type}*\mathrm{Run}\;\left|\;\text{Participant}\right.\right)+\left(1\;\left|\;\text{Electrode}\right.\right) \end{array}$$

**FIGURE 4:**
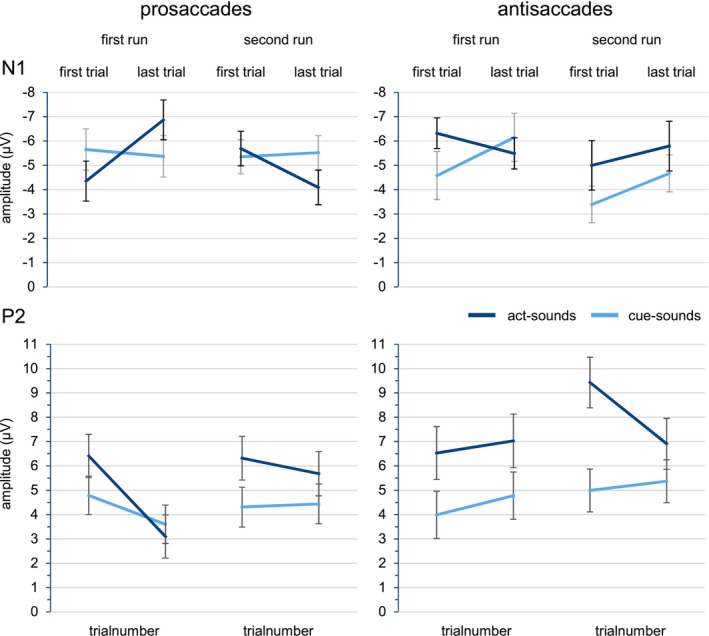
Line plots of the marginal estimated means for the linear mixed effects models. Lines represent the estimate over all 40 trials of each run. Error bars show one standard error at the start and end of each run, corresponding to the simple effects analyses.

R (Version 3.6.3) was used for statistical analysis, including the lme4 package (Version 1.1‐23) and lmerTest package (Version 3.1‐2) to test for significant effects with Satterthwaite approximated degrees of freedom. Significant interactions were examined by performing simple effects analyses. Interactions of categorical fixed effects (Sound Type, Saccade Type, Run) were examined by fitting two models that differed in their dummy‐coding (0, 1) of one involved predictor. The reference level was set to the first level in one model and to the second level in the other, and the remaining predictors (or interactions for multiple predictors) involved in the interaction were subsequently checked for significance. When resolving for the continuous predictor Trialnumber, we re‐centered it to the beginning (1) and end (40) of the run, similar to Volpert‐Esmond et al. (2021). Random effects were not changed for any simple effect analysis. An *α* level of 0.05 was considered statistically significant. Main effects are reported for the two experimental predictors, Sound Type and Saccade Type; interactions are only reported when they involve the predictor Sound Type. Full results are included in the R markdown file in addition to predictor coding and analyses at https://doi.org/10.17605/OSF.IO/BX8FU. Estimated marginal means of both models for the two components are visualized in the line plots in Figure 4.

#### 1.7.2 Behavioral Data

To examine behavioral differences between the pro‐ and antisaccade task, we separately fitted the reaction time, the interval between saccade offset and sound onset, and the number of errors (concerning saccade direction) data of the two act‐sound conditions to the following linear mixed models:

$$\text{Reaction time}\sim \text{Saccade type}+\left(1+\text{Saccade type}\;\left|\;\text{Participant}\right.\right)$$

$$\text{Saccade}-\text{Sound interval}\sim \text{Saccade type}+\left(1+\text{Saccade type}\;\left|\;\text{Participant}\right.\right)$$

$$\text{Number of errors}\sim \text{Saccade type}+\left(1\;\left|\;\text{Participant}\right.\right)$$

Analyzing the reaction time and saccade‐sound interval from single‐trial data allowed us to include the factor Saccade Type as a random effect. The number of errors was instead summed up for each saccade type, and thus aggregated. The reaction times of all correct trials were included in the analysis; for the saccade‐sound interval, we used all trials not excluded during the EEG preprocessing.

Differences in saccade execution between the act‐sound and act‐only conditions might indicate that the motor‐correction procedure used for the act‐only condition is not appropriate. To examine this, we separately fitted the peak speed and saccade amplitude values of saccades in trials remaining after all trial exclusion procedures in the EEG preprocessing to the following linear mixed model including the new predictor Action Type (act only [−0.5], act sound [0.5]):

$$\begin{array}{c} \text{Value}\sim \text{Action type}*\text{Saccade type}\\ \;+\left(1+\text{Action type}*\text{Saccade type}\;\left|\;\text{Participant}\right.\right) \end{array}$$

An *α* level of 0.05 was considered statistically significant for all analyses of the behavioral data.

## 2 Results

### 2.1 Behavioral Data

Descriptive statistics for the behavioral data can be found in Table 1. For reaction times, the model fit revealed a significant main effect of Saccade Type, *F*(1, 21.9) = 41.57, *p* < 0.001, with longer reaction times in the antisaccade task, *b* = 70.47. This main effect also reached significance for the number of saccadic errors, *F*(1, 22) = 36.72, *p* < 0.001, with more errors in the antisaccade task, *b* = 8.00.

**TABLE 1:** Descriptive statistics of behavioral data.

Behavioral data	Prosaccades		Antisaccades	
	*M*	SD	*M*	SD
Reaction time (ms)	292.1	128.5	363.9	93.2
Saccade errors	1.2	1.8	9.2	6.3
Sound onset	143.6	15.7	141.5	16.8

Saccade features	Act only				Act sound			
	Prosaccades		Antisaccades		Prosaccades		Antisaccades	
	*M*	SD	*M*	SD	*M*	SD	*M*	SD
Peak speed								
Run 1	317.3	62.9	314.8	65.7	316.9	64.2	319.4	64.0
Run 2	322.1	72.3	309.2	69.6	317.9	71.9	314.3	67.1
Amplitudes								
Run 1	9.7	1.2	10.0	1.5	9.6	1.3	10.0	1.5
Run 2	9.8	1.4	10.1	1.6	9.8	1.4	10.0	1.5
Overshoot								
Run 1	0.3	1.3	0.6	1.6	0.2	1.5	0.5	1.5
Run 2	0.3	1.4	0.5	1.6	0.3	1.5	0.5	1.6

*Note:* Means (*M*) and standard deviations (SD) for the behavioral data.

We found no significant main effect or interaction for saccadic peak speed, all *p*s > 0.163. For the saccade amplitude, we found a main effect of saccade type, *F*(1, 21.7) = 10.50, *p* = 0.004, with larger amplitudes for antisaccades, *b* = 0.34 (° visual angle), but no main effect of Action Type, *F*(1, 22.2) = 0.95, *p* = 0.339, and no interaction, *F*(1, 22) = 0.00, *p* = 0.968. To further investigate this pattern, we calculated how far saccades overshot the target dot on the *x*‐axis and analyzed this value with the same linear mixed model as the other saccade features. For the *x*‐axis overshoot, the model also revealed a main effect of Saccade Type, *F*(1, 21.6) = 9.37, *p* = 0.006, with a larger overshoot for antisaccades, *b* = 0.30 (° visual angle), and no main effect of Action Type, *F*(1, 22.2) = 1.19, *p* = 0.287, or interaction, *F*(1, 22.35) = 0.14, *p* = 0.709. As a consequence of the larger amplitude for antisaccades, the saccades had a longer duration, and the interval between saccade offset and sound onset showed a significant main effect of Saccade Type as well, *F*(1, 22) = 4.98, *p* = 0.036, with shorter intervals for antisaccades, *b* = −2.11.

### 2.2 N1 Component

There was no significant effect of Sound Type, *F*(1, 21.8) = 0.99, *p* = 0.330, Saccade Type, *F*(1, 21.02) = 0.65, *p* = 0.428, or a Sound Type by Saccade Type interaction, *F*(1, 21.9) = 1.69, *p* = 0.207. Instead, the model fit revealed a significant Sound Type by Saccade Type by Trialnumber three‐way interaction, *F*(1, 17,735) = 4.35, *p* = 0.037. Simple effect analysis showed that when resolving by Saccade Type, there was no underlying Sound Type by Trialnumber interaction for prosaccades, *t*(17,710.6) = −0.82, *p* = 0.415, only for antisaccades, *t*(17,712.1) = 2.07, *p* = 0.038. A resolution by Trialnumber revealed increased amplitudes for antisaccade act sounds compared to cue sounds at the start of runs, *t*(38.3) = −2.42, *p* = 0.020, *b* = −1.68, but not the end, *t*(39.8) = 0.34, *p* = 0.738. The resolution by Sound Type showed increasing N1 amplitudes over time for cue sounds, *t*(17,714) = −3.00, *p* = 0.003, *b* = −0.04 (1.46 μV over 40 trials), but not act sounds, *t*(17,680) = 0.03, *p* = 0.974.

The Sound Type by Saccade Type by Run by Trialnumber four‐way interaction reached significance as well, *F*(1, 17,739.6) = 12.03, *p* < 0.001, and a first resolution by Saccade Type showed that the underlying three‐way interaction was only significant for prosaccades, *t*(17,709.7) = 3.65, *p* < 0.001, and not antisaccades, *t*(17,716.8) = −1.38, *p* = 0.168. Instead, for antisaccades, only the significant Sound Type by Trialnumber interaction that was resolved above was found.

Resolving the Sound Type by Run by Trialnumber interaction for prosaccades revealed a significant Sound Type by Trialnumber interaction in the first run, *t*(17,701.7) = −3.18, *p* = 0.001, as well as the second run, *t*(17,707.8) = 1.99, *p* = 0.047. Further simple effects analyses revealed a trend for increased amplitudes for act sounds at the end of the first run, *t*(46.6) = −1.90, *p* = 0.064, *b* = −1.50, but not at the beginning, *t*(45.7) = 1.66, *p* = 0.105. The alternative resolution showed increasing amplitudes for act‐sounds, *t*(17,699.5) = −3.94, *p* < 0.001, *b* = −0.06 (−2.58 μV over 40 trials), but not cue sounds, *t*(17,697.1) = 0.46, *p* = 0.644. In the second run, there was also no change over time for cue sounds, *t*(17,693.1) = 0.29, *p* = 0.775, but here act‐sound amplitudes instead decreased significantly, *t*(17,697.1) = 2.44, *p* = 0.015, *b* = 0.04 (1.65 μV over 40 trials). Resolving the interaction by Trialnumber revealed no Sound Type effect at the beginning of the second run, *t*(42.6) = 0.42, *p* = 0.677, but a trend for attenuated act‐sound amplitudes at the end of the run, *t*(43) = 1.76, *p* = 0.086, *b* = 1.44, which is also visible in the marginal estimates means (see Figure 4). The remaining interactions including Sound Type did not reach significance, all *p*s > 0.155.

### 2.3 P2 Component

The model fit revealed a significant main effect of Sound Type, *F*(1, 21.9) = 24.82, *p* < 0.001, with higher amplitudes for act‐ compared to cue sounds, *b* = 1.89. The main effect of Saccade Type also reached significance, *F*(1, 21.9) = 7.04, *p* = 0.015, and the parameter estimate indicated higher amplitudes for antisaccade‐ compared to prosaccade‐generated sounds, *b* = 1.29.

We also found a significant Sound Type by Trialnumber interaction, *F*(1, 17,726.4) = 9.26, *p* = 0.002, and the resolution showed significantly decreasing amplitudes over time for act sounds, *t*(17,720.7) = −4.09, *p* < 0.001, *b* = −0.04 (−1.53 μV over 40 trials), but not cue sounds, *t*(17,718.2) = 0.08, *p* = 0.940. Despite this, the alternative resolution revealed increased act‐sound amplitudes at the start, *t*(44.5) = 5.85, *p* < 0.001, *b* = 2.65, and end of blocks, *t*(45.6) = 2.48, *p* = 0.017, *b* = 1.13.

Additionally, there was a significant Sound Type by Saccade Type by Run by Trialnumber four‐way interaction, *F*(1, 17,729.83) = 3.96, *p* = 0.047. Resolving this interaction by Sound Type, simple effects analyses showed a three‐way Saccade Type by Run by Trialnumber interaction for act sounds, *t*(17,729.2) = −3.91, *p* < 0.001, but not cue sounds, *t*(17,720.3) = −1.27, *p* = 0.205. Follow‐up analyses for act sounds then revealed a Run by Trialnumber interaction for prosaccades, *t*(17,710.6) = 2.72, *p* = 0.006, as well as antisaccades, *t*(17,700.6) = −2.80, *p* = 0.005, both of which can clearly be seen in Figure 4.

The prosaccade interaction was resolved to show significantly decreasing act‐sound amplitudes in the first run, *t*(17,697.7) = −4.84, *p* < 0.001, *b* = −0.09 (−3.40 μV over 40 trials), but not the second run, *t*(17,695.7) = −0.91, *p* = 0.364. For antisaccades, this was reversed, with significantly decreasing act‐sound amplitudes in the second, *t*(17,550.6) = −3.26, *p* = 0.001, *b* = −0.06 (−2.59 μV over 40 trials), but not the first run, *t*(17,700.2) = 0.67, *p* = 0.503. The respective Trial number effects for cue sounds, for which no significant three‐way interaction was found, showed no significant change over time, all *p*s > 0.064.

## 3 Discussion

In this study, we compared the neurophysiological sensory attenuation effects for self‐generated auditory stimuli, reflected in the N1 and P2 amplitudes, that were produced by pro‐ or antisaccades in order to examine a possible influence of interfering efference copies on forward model predictions concerning sensory consequences of actions. Participants performed either visually guided prosaccades to a target or antisaccades in the opposite direction, for which efference copies might be disturbed because of suppressed automated prosaccades (Coe and Munoz 2017; Munoz and Everling 2004). ERPs in response to the saccade‐generated sounds were compared to those for visually cued externally generated sounds. Mixed‐effect modeling of single‐trial data revealed a temporal dynamic of N1 amplitudes over the course of the experiment for prosaccade‐generated sounds, with increasing amplitudes in the first half of the experiment and decreasing amplitudes in the second. The N1 amplitudes of antisaccade‐generated sounds, however, did not change over time. A sensory attenuation effect in the sense of reduced N1 amplitudes for saccade‐generated relative to visually cued sounds was only indicated for prosaccades at the end of the experiment, where simple effects analysis for significant interactions revealed a trend in this direction. For the P2, we also found differing patterns for the two saccade types. While prosaccade‐generated sound amplitudes decreased significantly in the first but not the second block, this was reversed for antisaccade‐generated sounds, with a significant decrease only in the second block. Considering the identical temporal predictability for both sound eliciting actions, the results could demonstrate an influence of a disturbed or conflicting efference copy signal on the N1 and the P2.

### 3.1 N1 Component

Based on the findings by Mifsud et al. (2016) we expected an N1 attenuation for prosaccade‐generated sounds compared to cued externally generated sounds, and a diminished or missing attenuation for antisaccade‐generated sounds. This hypothesis could not clearly be confirmed, as we did not find a significant interaction between Sound Type and Saccade Type. Moreover, we expected different developments of N1 amplitudes over time for sounds following pro‐ and antisaccades, and this was indeed reflected in a four‐way interaction of both experimental predictors with the two predictors encoding the course of time during the experiment. Using the two‐level factor Run and the continuous factor Trialnumber (within Run) we found differing temporal dynamics of N1 amplitudes for pro‐ and antisaccades. While antisaccade‐generated sound amplitudes did not change over time, prosaccade‐generated sounds increased over the first run and decreased over the second run, as can be very well seen in Figure 4. Despite the significant decrease over time for prosaccade‐generated sounds in the second run, the cue‐sound to act‐sound difference at the end of the second run only revealed a trend for an N1 attenuation. Nevertheless, the pattern indicated at the end of the experiment, when the attenuation effect should be fully established, can be considered to be in line with our hypothesis to find a stronger attenuation effect for pro‐ than antisaccades.

The indicated N1 attenuation for sounds elicited by prosaccades is in line with the result by Mifsud et al. (2016). However, the effect might have been weakened by our use of visually cued externally generated sounds, compared to the uncued externally generated sounds employed by Mifsud et al. (2016). The prevalent account for the N1 attenuation is that sensory predictions are generated by feedforward models employing efference copy information right after motor planning to enable the early matching of predictions with actual sensory input, resulting in an attenuation of the N1 (Blakemore et al. 2001; Horváth 2015; Pickering and Clark 2014). Alternatively, this attenuation has been considered an effect of temporal predictability, which is inherent in self‐generated stimuli compared to unpredictable externally generated stimuli. Although predictability of sounds has been reported to lower N1 amplitudes (Harrison et al. 2021; Kaiser and Schütz‐Bosbach 2018), and a complete matching of overall temporal predictability between saccade‐generated and visually cued sounds was not possible, we do assume that the results of the present study cannot be ascribed to general predictability effects. That is because the comparison of sounds following pro‐ and antisaccades cannot be affected by temporal predictability, as their motoric execution (and their predictability) is comparable. Thus, the differing patterns for the N1 for which the amplitudes only for pro‐ but not for antisaccade‐generated sounds decreased toward the end of the experiment to a level below that of cued sounds indicates an effect of motor planning instead of predictability. As we have suspected, this might be an effect of the suppression of an automated prosaccade in the antisaccade condition (Coe and Munoz 2017), that leads to conflicting efference copy signals. In this way, our result suggests an involvement of motoric signals in the mechanism attenuating the N1 amplitude that is unrelated to the reduction ascribed to temporal predictability.

The fact that this attenuation (for prosaccades) was only found at the end of the experiment could be interpreted as a tuning of the feedforward model to establish the novel action–effect contingency of eliciting sounds by saccadic movement. Dogge et al. (2019) questioned the involvement of forward models in the N1 amplitude reduction, because their tuning should take longer than the short training sessions that are typically applied. In previous studies employing button press‐elicited sounds, however, the acquisition of an action–effect contingency might have been accelerated because of the common occurrence of button press‐elicited sounds in everyday life. For saccades, such experience in everyday life is unlikely, as they usually do not elicit sounds. Our results, and especially the specific changes in N1 amplitudes over time for saccade‐generated sounds, might thus be evidence for the slow tuning process of forward models for novel action–effect contingencies, as suggested by Dogge et al. (2019). This would also be in line with our previous report of a similar temporal dynamic of decreasing N1 amplitudes for sounds generated by observed actions from a first‐person perspective, which is also not commonly occurring in everyday life (Seidel et al. 2023). For third‐person observation, which is well trained on the other hand, no such temporal dynamic was found, and N1 amplitudes were attenuated from the start. The results of both studies suggest that action–effect associations that resemble those from everyday life and are thus familiar can be established more quickly than completely novel associations.

There are two patterns in the results for the N1, however, that raise questions about which contingencies were learned by participants in the different conditions. First, the prosaccade‐generated sounds show a large amplitude increase over time in the first run. Second, while amplitudes for visually cued sounds did not change over time in the prosaccade blocks, they increased significantly in antisaccade blocks. Considering the expectedly larger error rate in the antisaccade task, with sound omissions for saccades in the incorrect direction, the cued sound condition in the antisaccade block included trials in which visual cues were not followed by a sound as well. The learning of this lower contingency between cues and sounds could have led to a diminishing predictability of sounds expressed in increasing N1 amplitudes. This effect has likely been increased by the strict alternation between pro‐ and antisaccade blocks, as the previous visually cued condition contained a stronger contingency. The strong increase of the N1 amplitude for prosaccade‐generated sounds only during the first run might be directly connected to this larger error rate. It is possible that the very low error rate without sound omissions in the prosaccade task led over time to the impression that sounds are not actually saccade‐generated, but only visually cued. The higher error rate in the antisaccade task, which led to more demonstrations that sounds are only elicited when saccades are performed correctly, might have corrected this misinterpretation.

### 3.2 P2 Component

For the P2 we did not expect to find an amplitude reduction, neither for pro‐, nor for antisaccade‐generated sounds compared to external sounds, as reported by Mifsud et al. (2016). Instead, we found P2 amplitudes for saccade‐generated sounds to be generally enhanced, but with slightly differing temporal dynamics for the saccade types. For prosaccades, act‐sound P2 amplitudes decreased in the first run, but for antisaccades, they decreased in the second run. The general enhancement, which was also represented by a main effect of the Sound Type, can be explained by the fact that amplitudes were compared with those for visually cued sounds, for which reduced P2 amplitudes have been shown (Sowman et al. 2012), while Mifsud et al. (2016) used uncued external sounds in the comparison condition for saccade‐generated sounds.

Because the P2 amplitude attenuation has been associated with more contextual influences that are independent of the motor‐based forward models (Knolle et al. 2013; Seidel et al. 2021; Sowman et al. 2012; Timm et al. 2016), we did not expect to find differing patterns for pro‐ and antisaccade‐generated sounds, which only vary in their motor planning. But while P2 amplitudes for prosaccade‐generated sounds decreased already over the first run to a level below that of cued sounds, antisaccade‐generated sounds only decreased toward the end of the experiment to a level of cued sounds, as can be seen in Figure 4. Considering previous results showing increased P2 amplitudes under a perceived loss of agency or control over sound production (Seidel et al. 2021; Timm et al. 2016), this might indicate a diminished belief to generate these sounds. The earlier decrease of the P2 for prosaccade‐generated sounds could thus be interpreted as a faster increase of the level of agency or control over the production of sounds than for antisaccades, for which P2 amplitudes are only decreased toward the end of the experiment. Such a difference in agency between pro‐ and antisaccades might stem from the lack of experience in performing antisaccades in everyday life. It is possible that the subjective feeling of having performed an antisaccade is diminished by the strong top‐down regulation used to suppress the automated prosaccade that one intuitively wants to perform. The conflicting efference copy signals that we expected for antisaccades and that may be reflected in the result pattern for the N1 might also hinder a clear agency assignment in this condition.

### 3.3 Limitations

A central problem in the comparison of the pro and antisaccade blocks is the larger amount of error trials for antisaccades, which not only reduces the total amount of trials in which the action‐sound contingency can be experienced, but also influences the playback of visual cues and sounds in the cued sound control condition. As we have discussed above, the increase of N1 amplitudes over time for cue‐sound amplitudes in the antisaccade blocks might be explained by this, and future studies should consider opting for randomized sound timings and a stable cue‐sound contingency instead of exact replays. However, it is unclear whether this is an issue for the antisaccade‐generated sounds, as the action–effect contingency between saccades performed in the task‐compliant direction and sounds should have been trained in all blocks, independently of the saccade type.

On the other hand, we implemented harsher than anticipated criteria to include trials in our analysis by making sure that appropriate saccades were executed and that saccades were properly matched between conditions. This resulted in the exclusion of fifteen participants overall and reduced the sample size considerably compared to Mifsud et al. (2016) to examine saccade‐generated ERP amplitudes. This may have led to reduced power in our analysis, which may explain some of the negative findings and may also provide an explanation for the trends we found in the last step to resolve the four‐way interaction found for the N1 amplitudes.

Finally, we did not systematically track potential eye movement during the cue‐conditions (cue sound and cue only), in which participants were asked to not perform a saccade and only fixate the visual cue. An exploratory analysis of the horizontal electrooculogram (hEOG) that was assessed as part of the EEG acquisition did, however, not indicate that the participants performed horizontal saccades during the cue‐conditions (see Supporting Information S4).

## 4 Conclusions

This study provides evidence for the integration of motoric signals in the formation of predictions for auditory action effects, by examining N1 and P2 amplitudes for sounds generated by prosaccades and antisaccades in comparison to cued external sounds. Mixed linear model analysis on trial‐level data revealed an N1 reduction for prosaccade‐generated sounds, but not for antisaccade‐generated sounds, that developed toward the end of the experiment. This reduction is in line with findings for button press‐elicited sounds, but the slow temporal dynamic might indicate that unusual action–effect contingencies, like sounds generated by saccades, might involve a slower tuning process of internal forward models, while only fine‐tuning is necessary when similar action effects are encountered in everyday life, such as sounds following button presses. For antisaccade‐generated sounds, the missing N1 reduction might hint at conflicting efference copy information relayed to predictive models, as antisaccades require the suppression of automatically generated prosaccades. We also report a P2 for prosaccade‐generated sounds that decreased faster than that for antisaccade‐generated sounds. This is potentially an effect of reduced perceived control or agency over sound generation when performing antisaccades.

## Author Contributions

**Alexander Seidel:** conceptualization, data curation, formal analysis, investigation, methodology, software, visualization, writing – original draft, writing – review and editing. **Christian Bellebaum:** project administration, resources, supervision, writing – review and editing.

## Conflicts of Interest

The authors declare no conflicts of interest.

## Supporting information


Data S1.

## Data Availability

Raw data cannot be shared due to ethics regulations for this study, model output files are openly available at https://doi.org/10.17605/OSF.IO/BX8FU.
