# Supplementary material for: Conflicting Motor Plans and Sensory Attenuation: Evidence From Event‐Related Potentials for Sounds Generated by Pro‐ and Antisaccades
Source: Psychophysiology. 2025 Jul 30;62(8):e70114. doi: 10.1111/psyp.70114 (PMC12308631; doi:10.1111/psyp.70114)
Supplement: Supplementary file 1 — Data S1. [file PSYP-62-e70114-s001.docx]

Supplementary material to:

**Conflicting motor plans and sensory attenuation: Evidence from event-related potentials for sounds generated by pro- and antisaccades**

Alexander Seidel, Christian Bellebaum

Faculty of Mathematics and Natural Sciences,

Heinrich Heine University Düsseldorf, Germany

Running head: Auditory ERP attenuation after pro and antisaccades

*Corresponding author*: Alexander Seidel, Heinrich Heine University, Faculty of Mathematics and Natural Sciences, Universitätstrasse, 1, 40255, Düsseldorf, Germany.

Email: [Alexander.Seidel@outlook.com](mailto:Alexander.Seidel@outlook.com)

**
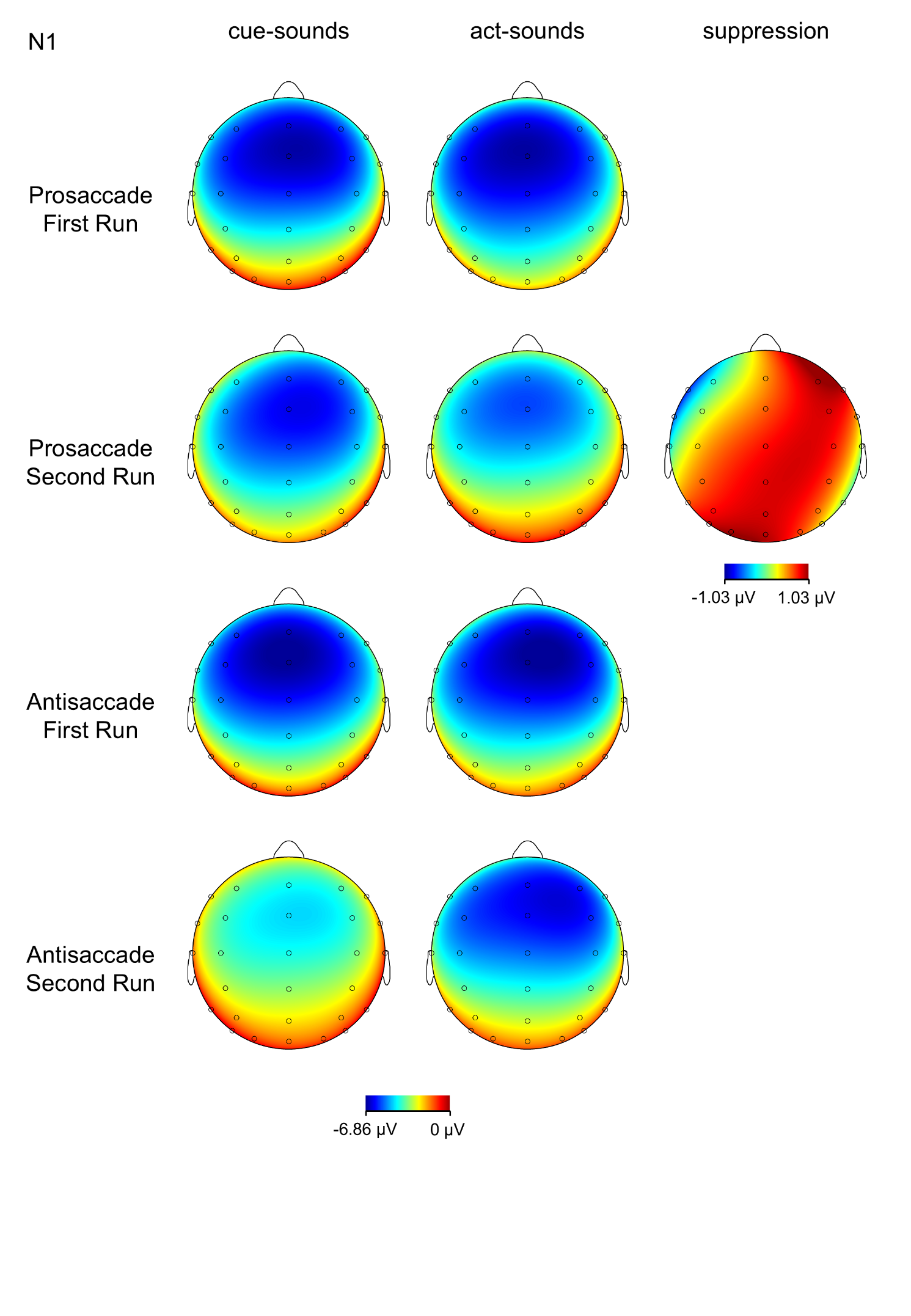
S1. N1 Topographies**

*Note*. Topographical maps showing scalp potentials at the time of the N1 peaks from the grand average ERPs seen in Figure 3, including the N1 suppression for prosaccade-generated act-sounds in the second run.

**
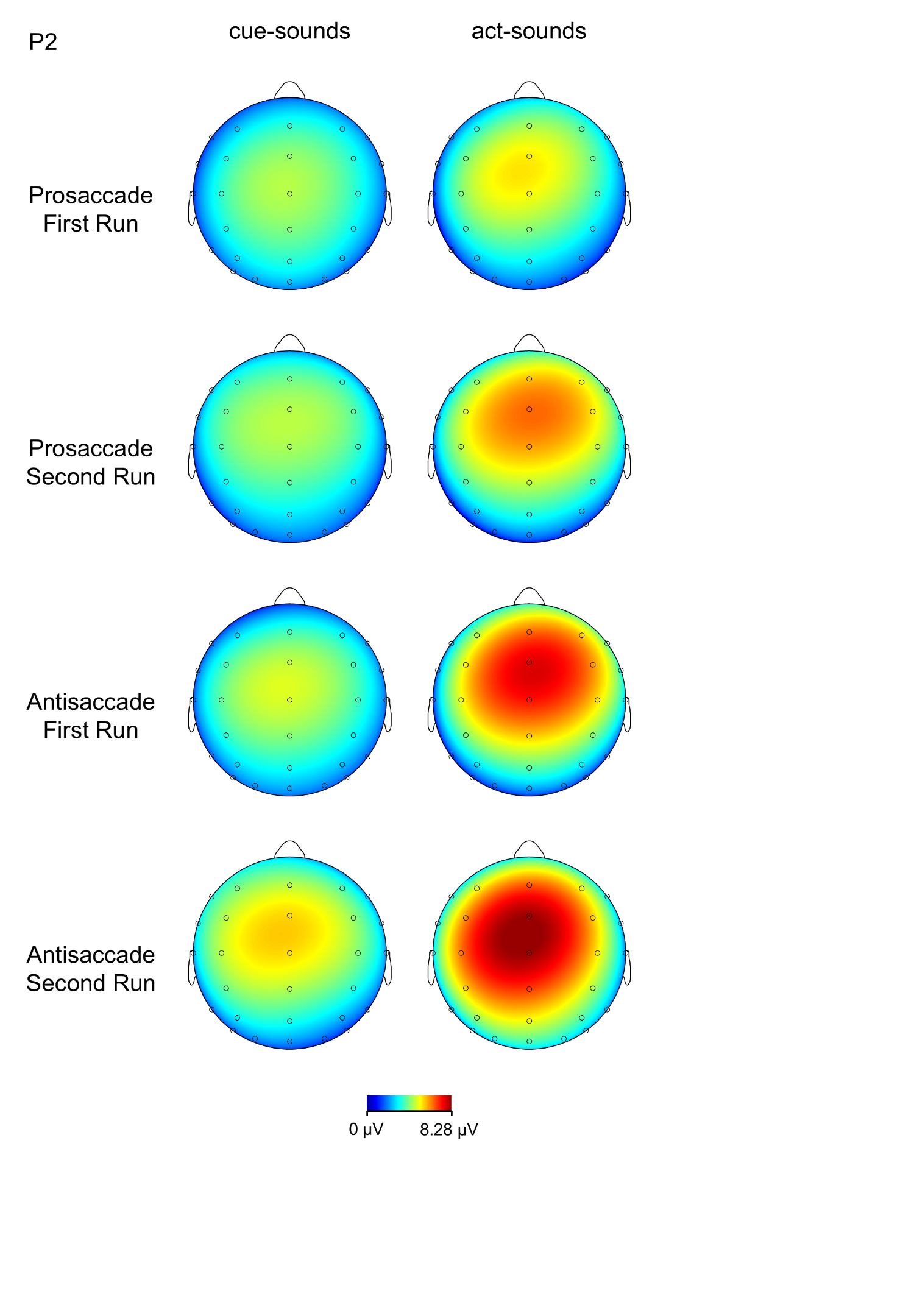
S2. P2 Topographies**

*Note*. Topographical maps showing scalp potentials at the time of the P2 peaks from the grand average ERPs seen in Figure 3. The suppression column is omitted, as no P2 suppression for act-sounds was found.

**S3. Analysis of peak latency data**

To identify individual time windows to extract mean amplitude values in each trial, individual peaks in the average ERP signal (pooled over FZ, FCz and Cz) of each sound type (act-sound, cue-sound), saccade type (prosaccade, antisaccade), run (first run, second run) and participants were determined. Here we show an analysis following the same methodology as the main analysis for these peak latency values for the N1 and P2 peaks, using the above predictors in the here specified linear mixed model:

$$Latency \sim Sound Type*Saccade Type*Run+ \left( 1\left| \mathrm{Participant} \right. \right)$$

No main effect or interaction reached significance for the N1 (all *p*s > .214) or P2 (all *p*s > .167) latency values. The full output files can be found at: <https://doi.org/10.17605/OSF.IO/BX8FU>

**S4. Analyses of the horizontal electrooculogram data in the cue conditions**

For an exploratory analysis of saccadic movements during the cue conditions, we checked the horizontal electrooculography (hEOG) data. More specifically, we analyzed the data of the hEOG-electrode for the cue conditions and the act-sound conditions in the same way to see whether typical saccade-related hEOG signals from the saccade conditions could be found in the cue conditions as well. We first calculated the difference between the minimum and maximum amplitude value in the time window from -200 to -100ms before sound onset, in which saccades typically appeared in the saccade conditions, in each trial of the act-sound condition for each participant, and then determined the average amplitude difference for each participant/saccade type/run combination. For each cue-sound and cue-only segment we then checked in each trial whether the max-min amplitude difference reached at least 70% of the magnitude of the average act-sound difference of the corresponding participant/saccade type/run combination. Over all participants, this only revealed a mean of 1.16 trials (SD = 2.13) per run of 40 trials in which the 70% limit was exceeded, indicating that there were no signs of saccade-related activity in the cue condition.
